# Co-Treatment with Ritonavir or Sertraline Enhances Itraconazole Efficacy Against Azole-Resistant *Trichophyton indotineae* Isolates

**DOI:** 10.3390/jof11100698

**Published:** 2025-09-25

**Authors:** Anna Günther, Anke Burmester, Mario Fabri, Jörg Tittelbach, Cornelia Wiegand

**Affiliations:** Department of Dermatology, Jena University Hospital, Friedrich Schiller University, D-07747 Jena, Germanyjoerg.tittelbach@med.uni-jena.de (J.T.); c.wiegand@med.uni-jena.de (C.W.)

**Keywords:** antifungal resistance, itraconazole, ritonavir, sertraline, *Trichophyton*, *indotineae*, transporter inhibitor

## Abstract

The treatment of azole-resistant *Trichophyton indotineae* poses a significant challenge for clinicians worldwide. Resistance mechanisms include amino acid substitutions in the sterol 14-α demethylase gene *Erg11B*, as well as overexpression of *Erg11B*. Additionally, efflux mechanisms mediated by fungal transporter proteins contribute to antifungal resistance. Therefore, the inhibition of fungal efflux transporters using known inhibitors could be a promising strategy to prevent treatment failure. The inhibitory effects of itraconazole in combination with various efflux pump inhibitors were evaluated. Co-treatment with quinine hydrochloride and itraconazole did not lead to a significant reduction in the inhibitory concentration (IC) values in *T. indotineae* isolates. In contrast, ritonavir lowered IC values by approximately 50% without affecting fungal growth when applied as monotherapy. The most pronounced effect was observed with sertraline, which demonstrated intrinsic antifungal activity at higher concentrations. When combined with itraconazole, sertraline reduced IC values to below 10% in both susceptible and resistant strains, enhancing itraconazole efficacy markedly. The increasing prevalence of antifungal resistance is a growing global health concern. These findings suggest that sertraline holds considerable potential as an adjunctive therapy for the treatment of dermatomycoses.

## 1. Introduction

Recalcitrant dermatomycoses have emerged as a growing public health problem and continue to pose a challenge for both the diagnosis and treatment of affected patients reviewed in [1]. In the past decade, the number of resistant *Trichophyton* isolates has markedly increased, necessitating the expansion of diagnostic protocols and the adaption of treatment strategies [2,3,4]. One major contributor to the rise in resistant isolates is the emergence of a novel genotype, designated as ITS type VIII within the *Trichophyton mentagrophytes*/*interdigitale* complex. These isolates have been associated with a widespread epidemic of recalcitrant dermatomycosis in India [5,6,7,8]. A high proportion of these isolates exhibited terbinafine resistance, predominantly due to point mutations that alter the amino acid sequence of the squalene epoxidase gene *Erg1* [5,6,7,9]. Phylogenetic analyses based on whole-genome sequencing revealed that these isolates form a basal lineage within the *T. mentagrophytes*/*interdigitale* complex [10]. Consequently, a new species designation, *Trichophyton indotineae,* was recently proposed [11,12]. The absence of mating compatibility with strains of the opposite mating type supported the classification of *T. indotineae* as a distinct species [11,12]. In addition to terbinafine resistance, several *T. indotineae* isolates also displayed reduced susceptibility to itraconazole and voriconazole [5,9,13]. Azoles target the sterol 14-α demethylases, which belong to the cytochrome p450 superfamily (*CYP51*), referred to as *Erg11* following the *Candida* nomenclature. Two *Erg11* paralogs have been identified in *T. indotineae* [14]. Altered amino acid sequences associated with azole resistance have been found exclusively in the *Erg11B* gene of *T. indotineae* [14,15,16]. Genomic analyses further revealed that *Erg11B* is duplicated in several strains, resulting in two distinct types of genomic amplification [15,17]. Type I includes the tandem amplification of a 2.4 KB fragment of *Erg11B* [15], whereas type II encompasses the amplification of a 7.4 KB fragment of *Erg11B* and neighboring genes [17]. Both amplification types lead to *Erg11B* overexpression, which has been associated with reduced azole susceptibility [15,17]. *Erg11B* overexpression was, for instance, confirmed for the azole-resistant *T. indotineae* isolate UKJ 476/21 [18], which belongs to the type II amplification group [19]. Efflux-mediated antifungal resistance also contributes to treatment failure. The overexpression of the ATP-binding cassette (ABC) transporter with homology to the multidrug resistance (MDR) transporter has been described, initially for *Trichophyton rubrum MDR3* [20]. *MDR3* overexpression was later also detected in the *T. indotineae* strains TIMM20118 and TIMM20119 [15]. However, itraconazole resistance can occur independently of *MDR3* expression; *Erg11B* overexpression alone is sufficient to confer resistance [15]. Interestingly, exposure to fluconazole induced *MDR3* expression in azole-susceptible *T. indotineae* strains [18].

Antifungal resistance is not always attributable to genetic alterations in drug target genes. For instance, terbinafine resistance has been observed in some *T. mentagrophytes* strains that retained wild-type *Erg1* sequences [21]. Similarly, treatment failure in terbinafine-treated *T. indotineae* infections occurred despite isolates carrying wild-type *Erg1* genes [22]. In the case of azole resistance in *T. rubrum,* mutations in *Erg11B* were only detected in a subset of the resistant isolates [19,23]. Therefore, molecular diagnostics targeting known resistance genes should be complemented by antifungal susceptibility assays to ensure accurate resistance profiling. Several standardized antifungal susceptibility tests utilizing broth microdilution protocols have been developed by the EUCAST or CLSI committees [24,25,26]. In addition, simplified plate-based assays have been proposed to reduce time and resource demands [27]. Nevertheless, EUCAST or CLSI protocols remain the gold standard due to their higher accuracy and reproducibility. These assays specify standardized parameters such as inoculum size, spore concentration, incubation temperature, and a two-fold (1:2) serial dilution range for antifungal agents [24,25,26]. Readouts can be assessed visually or via a spectrophotometer [24,25,26]. Microplate laser nephelometry (MLN) provides an automated method for monitoring fungal growth dynamics during antifungal susceptibility testing. The method has been validated for multiple fungal species [28], including *T. indotineae* [14] and *T. quinckeanum* [29]. EUCAST and CLSI protocols have been adapted for use with MLN to evaluate the influence of media composition, incubation temperature, and spore concentration for inoculation on itraconazole inhibitory concentrations (ICs) [19]. An optimized MLN protocol was subsequently applied to analyze the effects of co-treatment with potential efflux pump inhibitors, assessing synergistic interactions with itraconazole.

Pharmaceuticals were based on their known activity against human ABC transporters as well as their reported antifungal properties. Quinine, derived from the bark of the Cinchona tree, is classified as a first-generation inhibitor of the human MDR1 transporter [30]. Ritonavir and sertraline are both substrates of the human transporter P-gp [30]. All compounds have also been shown to exhibit antifungal activity, which may be linked to analogous effects on fungal efflux transporters. Quinine has been reported to inhibit yeast-to-hyphae morphogenesis and biofilm formation in *Candida albicans* [31]. Ritonavir demonstrated antifungal activity against *Histoplasma capsulatum* [32] and *Candida auris* [33]. While ritonavir alone was able to sufficiently repress fungal growth in *H. capsulatum* [32], it only displayed antifungal activity against *Candida auris* when combined with azoles [33]. Among these compounds, sertraline has been most extensively studied for its antifungal properties. Clinical evidence demonstrated that patients suffering from recurrent vulvovaginal candidiasis were successfully treated with oral sertraline at 50 mg over 5–8 menstrual cycles [34]. MIC_99_ values ranging from 3 to 29 µg/mL were reported for *C. albicans*, *C. glabrata,* and *C. parapsilosis* isolates causative for the infection [34]. Other isolates of *C. albicans* and *C. glabrata* exhibited lower susceptibility against sertraline, with MIC values between 32 and 128 µg/mL [35]. For these less susceptible strains, synergistic effects were observed when sertraline was combined with essential oils from *Cinnamomum verum,* resulting in an approximate 90% MIC reduction for both compounds [35]. In vitro studies further showed that treatment with sertraline or fluoxetine reduced biofilm formation of *C. krusei* and *C. parapsilosis* [36]. Clinical isolates of *Cryptococcus neoformans* displayed in vitro MIC_90_ values of 2–6 µg/mL for sertraline, with additional synergistic effects observed when combined with fluconazole [37]. Moreover, sertraline exhibited specific activity against cryptococcal fungicide-tolerant persister cells [38]. The therapeutic potential of sertraline for life-threatening fungal infections has also stimulated the research of sertraline derivatives targeting ergosterol biosynthesis [39]. The fungicidal properties of sertraline were further demonstrated in *Trichosporon asahii*, a yeast belonging to the basidiomycota, with MIC_90_ values of 8 µg/mL [40]. In *Coccidioides immitis,* MIC values of 4–8 µg/mL were recorded, along with synergistic interactions when combined with fluconazole [41]. Finally, synergistic effects of sertraline with caspofungin have been proposed as a novel treatment approach for *Trichophyton rubrum* infections, particularly in cases refractory to standard antifungals [42].

Although *T. indotineae* infections are not life-threatening, they frequently affect large areas of the body, resulting in tinea corporis and tinea cruris. The majority of patients are young, otherwise healthy male adults. In accordance, the infection has severe consequences for social life, sports activities, and personal relationships. Treatment is increasingly challenging due to the high prevalence of terbinafine-resistant isolates. Moreover, a considerable proportion of isolates display multidrug resistance, involving terbinafine as well as several azoles. The primary goal of this study was to identify human-approved pharmaceuticals with intrinsic antifungal activity that could be repurposed in combination with first-line antifungals. Such combination strategies may provide novel therapeutic options and help to overcome current treatment failures in resistant *T. indotineae* infections.

## 2. Materials and Methods

### 2.1. Strains and Growth Conditions

Strains were obtained from routine cases at Jena University Hospital (UKJ) or from a previous study that collected strains from all parts of India [9]. Several strains were sent out to other groups and used for genomic analyses as listed in Table S1 [15,17]. Strains were pre-cultivated for three to eight weeks on Dermasel agar (Thermoscientific, Wesel, Germany) for MLN analysis. SG was prepared using Sabouraud-2% *w*/*v* glucose bouillon (Merck, Darmstadt, Germany) and used for liquid growth.

### 2.2. Type II and I Specific PCR, qPCR, and DNA Sequencing

For strain verification, the *ITS* region and squalene epoxidase gene *Erg1* were sequenced as described previously [8,9]. The sterol 14-α demethylase gene *Erg11B* was sequenced as described previously [14]. PCRs for type I or type II specific fragments were amplified as previously described [17,19]. Fragments were separated using agarose gel electrophoresis. The determination of *Erg11B* genomic copy numbers was performed with qPCR conditions as described for cDNA analysis [18]. Primers and calculations were performed as previously described [19]. CT values were normalized using UKJ 1676/17 as *Erg11B* single-copy control, as previously described [17]. The fold change was calculated using the 2^−ΔΔCT^ method [43]. The algorithm was corrected for primer efficiency as described [44]. Two biological replicates and two technical replicates were determined with qPCR.

### 2.3. Medical Stock Preparations and Concentration Ranges

Itraconazole was obtained from Sigma Aldrich, St. Louis, MO, USA, and solved in dimethyl sulfoxide (DMSO, Carl Roth, Karlsruhe, Germany). The concentration was determined as described using a spectrophotometer [45]. The stock concentrations ranged between 0.5 and 1 mg/mL. Quinine hydrochloride (Merck, Darmstadt, Germany) was dissolved in H2O for stocks of 0.4 mg/mL and sterilized using a sterile filter with a pore size of 0.2 µm. Ritonavir (Sigma Aldrich) was dissolved in 99% ethanol p.A. with a stock concentration of 7,21 mg/mL. Stocks were diluted to reach identical ethanol concentrations in MLN assays. MLN assays with ritonavir showed a final concentration of 0.5% *v*/*v* ethanol. Sertraline (Sigma Aldrich) was solved in DMSO as a stock of 20 mg/mL. Further dilutions were performed to keep the DMSO concentration constant. The final DMSO concentration of 0.25% *v*/*v* was used in MLN assays. Itraconazole in the range of 0.125 µg/mL up to 0.001 µg/mL was used for strains harboring *Erg11B* type II and I genomic amplifications. For azole-sensitive strains, the range starts from 0.03 µg/mL and ends at 0.0002 µg/mL.

### 2.4. Microplate Laser Nephelometry (MLN) Assays

MLN conditions were as previously described [29]. The conditions were adapted to the CLSI protocol [26]. The spore suspensions were adjusted to final concentrations of 3 × 10^3^ CFU/mL, and the growth temperature was set to 34 °C. Medium was changed into SG to enhance growth conditions as described previously [19]. For the minimization of physiological differences dependent on pre-cultivation or the viability of counted spores, strains were treated exclusively with itraconazole in every assay. The controls were used for normalization, and the obtained IC_50_ values were set as one. Therefore, the relation was determined between single-treated and co-medicated assays. For assays using ritonavir with the solvent ethanol, the effects of ethanol in combination with itraconazole were always determined. Growth controls were obtained from untreated cells, solvent-treated cells, and cells treated exclusively with co-medicals. Growth control of untreated cells was used for normalization and to determine the relation between control and other treatments depending on the solvent or co-medicals. Two biological and two technical replicates were obtained. The plates were measured every hour for a 120 h period.

### 2.5. Graphical Image Preparation, Data Performance, and Statistical Analysis

Graphical images were prepared using the OriginPro 2025 (Origin-Lab Corporation, Northampton, MA, USA) software. IC_50_ values were performed for non-linear curves using the logistic option with the Levenberg–Marquardt algorithm of OriginPro2025 as previously described [19]. Statistical analysis was performed with IBM SPSS Statistics 30. A pairwise Mann–Whitney U test was used to determine the asymptotic significance values.

## 3. Results

Several *T. indotineae* strains included in this study have been previously analyzed and were well-characterized with respect to their antifungal resistance profile [9,14,15,17,18]. For many of these strains, whole-genome sequence data are available [15,17] (see Table S1). With regard to itraconazole susceptibility, eight of the strains exhibited resistant phenotypes, while four strains were classified as susceptible. Detailed characteristics of all strains are summarized in Table 1. All strains exhibiting genomic amplification of *Erg11B* (Table 1) demonstrated itraconazole resistance. Strains harboring *Erg11B* double mutations without genomic amplification (Table 1) displayed an increased tolerance to sertaconazole nitrate [14]. Strains carrying the *Erg1* Phe397Leu substitution were highly resistant to terbinafine, whereas the *Erg1* Ala448Thr mutation was not associated with high terbinafine resistance but was found to co-occur with *Erg11B* type II amplification [17]. Although strains UKJ1067/21 and UKJ1985/21 retained wild-type *Erg1* sequences, they were isolated from a patient who experienced treatment failure with terbinafine [22].

### 3.1. Erg11B Copy Number Determination and Type II and I Specific PCR to Verify Strain Genotypes

**Figure 1:**
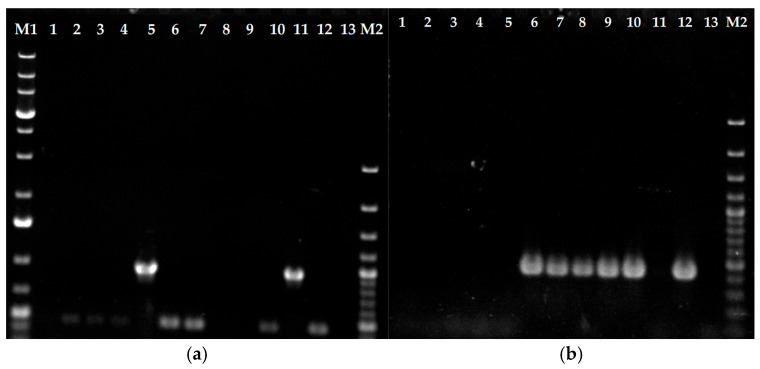
*Erg11B* type II (**b**) and I (**a**) genomic amplification-specific PCR. Genomic DNA from itraconazole-sensitive strains UKJ 1676/17, UKJ 262/21, UKJ 1067/21, and UKJ 1985/21 was loaded in lanes 1 to 4 of panels (**a**) and (**b**), respectively. PCR results for the itraconazole-resistant strains UKJ 1687/17, UKJ 1708/17, UKJ 392/18, UKJ 476/21, UKJ 334/19, UKJ 336/19, UKJ 421/18, and UKJ 893/19 are presented in the same order in lanes 5 to 12. Negative controls were included in lane 13. Molecular size markers were loaded in lanes M1 and M2: the 1 KB ladder Plus (Thermo Fisher Scientific, Vilnius, Lithuania) was used in lane M1 and the 100 bp Plus ladder (Thermo Fisher Scientific) in lane M2. Marker bands with three-fold DNA concentrations were visible at 0.5 KB and 1 KB for the 100 bp ladder Plus and at 0.5 KB, 1.5 KB, and 5 KB for 1 KB ladder Plus.

For verification of the genotype of *T. indotineae* strains, type I- and type II-specific PCR assays were performed according to a previously described protocol [17]. All strains assigned a TIMM collection number (see Table 1) displayed amplification patterns consistent with earlier reports [17] (Figure 1). However, a discrepancy was identified for strain UKJ1708/17, which displayed a type II amplification pattern, contrary to its previous classification as type I [17] (Figure 1b, lane 6). Type I amplifications were confirmed for strains UKJ1687/17 and UKJ421/18, both showing the expected 955 bp fragment (Figure 1a, lanes 5 and 11). Strain UKJ476/21 exhibited a type II-specific amplification fragment of 434 bp (Figure 1b, lane 8), which was accompanied by *Erg11B* overexpression, as previously described [18]. Type II-specific fragments were also detected in strains UKJ392/18, UKJ334/19, UKJ336/19, and UKJ893/19, confirming earlier results [17] (Figure 1b, lanes 7, 9, 10, and 12). UKJ262/21 displayed the same amplification pattern as UKJ1676/17 (Figure 1, lanes 1 and 2). Notably, this strain exhibited susceptibility to itraconazole, voriconazole, and sertaconazole nitrate [14].

**Figure 2:**
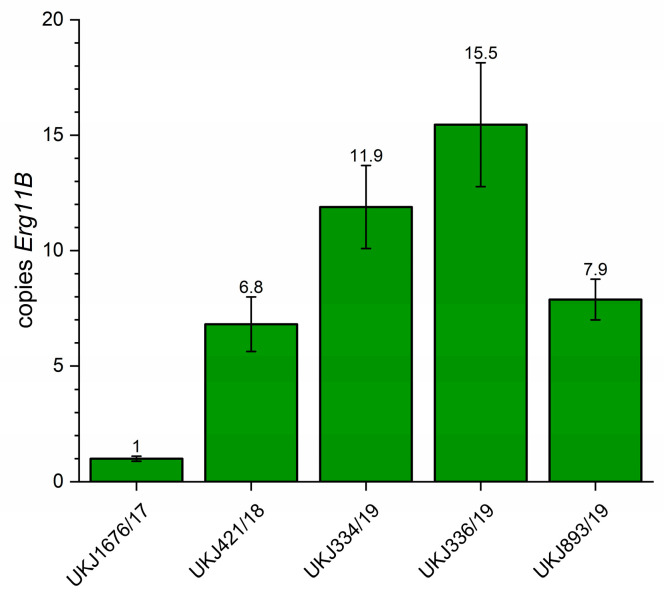
Determination of *Erg11B* copy number using quantitative real-time PCR. Strain UKJ1676/17, known to carry a single *Erg11B* copy, was used as the reference control. qPCR mean values obtained from this strain served as the normalization baseline for determining relative *Erg11B* copy numbers in other strains. The numbers displayed above each column represent the mean genomic *Erg11B* copy number for the respective strain. Error bars indicate variation in technical and biological replicates.

For the determination of the genomic copy number of *Erg11B* in *T. indotineae* strains exhibiting type I and II amplification patterns, quantitative real-time PCR (qPCR) was performed according to previously established protocols [17,19]. In type I strains, qPCR revealed five to seven *Erg11B* copies arranged in tandem repeats, consistent with results obtained from whole-genome data [17]. In contrast, qPCR-derived copy numbers for type II strains deviated from genome-based estimates [17], and notable variation was observed among individual type II isolates. The results presented in Figure 2 corroborate prior findings [17,19], confirming increased variability in *Erg11B* copy numbers within type II strains. Specifically, strain UKJ334/19 exhibited up to 12 copies, while UKJ336/19 showed a maximum of 15 copies (Figure 2), compared to typical copy numbers of 7–8 in type II strains [17,19]. Previously reported copy numbers for TIMM20120 (UKJ334/19: 9–11 copies) and TIMM20121 (UKJ336/19: 14 copies) [17] supported the correct designation of UKJ strains with TIMM isolates. Notably, UKJ 336/19 harbors a double mutation in *Erg1* (see Table 1), which correlates with high-level resistance to terbinafine.

### 3.2. Treatment of Itraconazole in Combination with Quinine Hydrochloride Showed a Weak Effect on Inhibitory Concentrations of 50% (IC_50_) of T. indotineae Strains

**Figure 3:**
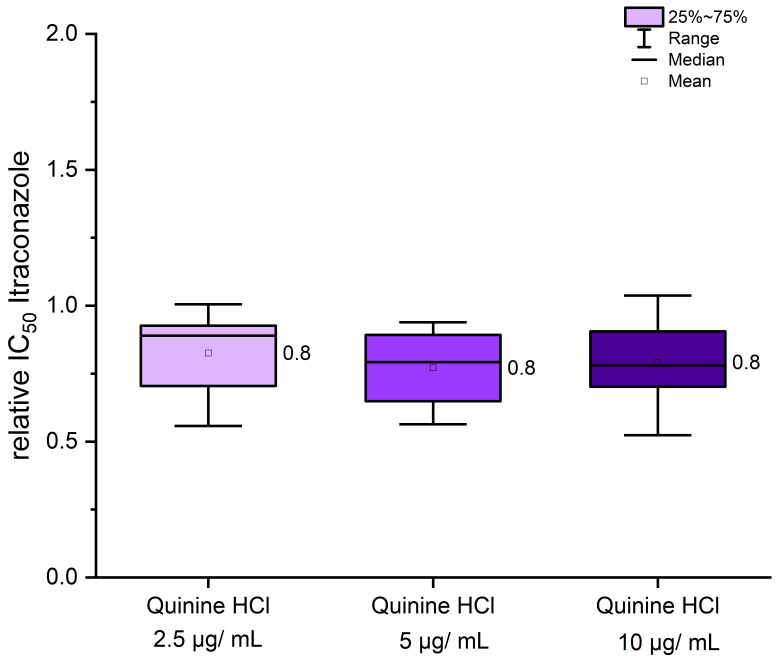
Co-treatment of itraconazole with quinine hydrochloride resulted in only a modest effect, which appeared to be independent of the strains’ genotypic background. IC_50_ values from itraconazole monotherapy were used for normalization (set as 1). Relative changes in IC_50_ values following co-treatment with quinine hydrochloride are presented as fold differences. Mean values are indicated next to the boxplots. A total of eight strains, as listed in Table S2, were included in the analysis.

Quinine hydrochloride is an inexpensive and widely available pharmaceutical compound that may offer potential in settings with limited healthcare resources. Its high water solubility presents an additional advantage for formulation and topical application. Microplate laser nephelometry (MLN), as previously described [11,19], was employed to determine the IC_50_ value (half-maximal inhibitory concentration) of itraconazole, both alone and in combination with quinine hydrochloride. Experimental parameters were adapted based on the CLSI protocol M38 [26]. Incubation was conducted at 34 °C, with a final spore titer of 3 × 10^3^ cfu/mL for each strain. Over a total incubation time of 120 h, MLN measurements were taken every hour. Instead of RPMI1640 medium (buffered with MOPS, pH 7.0), Sabouraud glucose (SG) medium containing 2% *w*/*v* glucose was used. As demonstrated in previous studies [19], the use of SG medium led to a significant decrease in IC_50_ values and allowed for better discrimination between azole-resistant or azole-susceptible strains [19]. To detect even small effects, IC_50_ values for itraconazole alone were always determined as baseline control, thereby minimizing variability due to strain-specific pre-cultivation conditions. In Figure 3, the ratio of IC_50_ values from itraconazole monotherapy to those from co-treatment with quinine hydrochloride is presented. Effective co-treatment is indicated by a decrease in IC_50_ values relative to the itraconazole control. Increasing concentrations of quinine hydrochloride beyond a certain threshold achieved no additional effect, and a final concentration of 2.5 µg/mL was sufficient to reduce IC_50_ values by approximately 20% (Figure 3), from 0.081 µg/mL in the itraconazole monotherapy to around 0.068 µg/mL in the co-treatment of UKJ1708, for example. However, the degree of IC_50_ reduction varied among strains, as shown in Table S2. Importantly, these strain-specific responses did not correlate with specific resistance genotypes (see Table 1). Notably, certain strains, such as UKJ1676/17, UKJ1708/17, and UKJ 476/21, showed no measurable effect during quinine hydrochloride co-treatment (Table S2). Due to the modest efficacy of quinine hydrochloride, only a subset of strains was tested, and further analyses were limited.

### 3.3. Co-Treatment with Ritonavir Showed a 50% Reduction in Itraconazole IC_50_ Values

**Figure 4:**
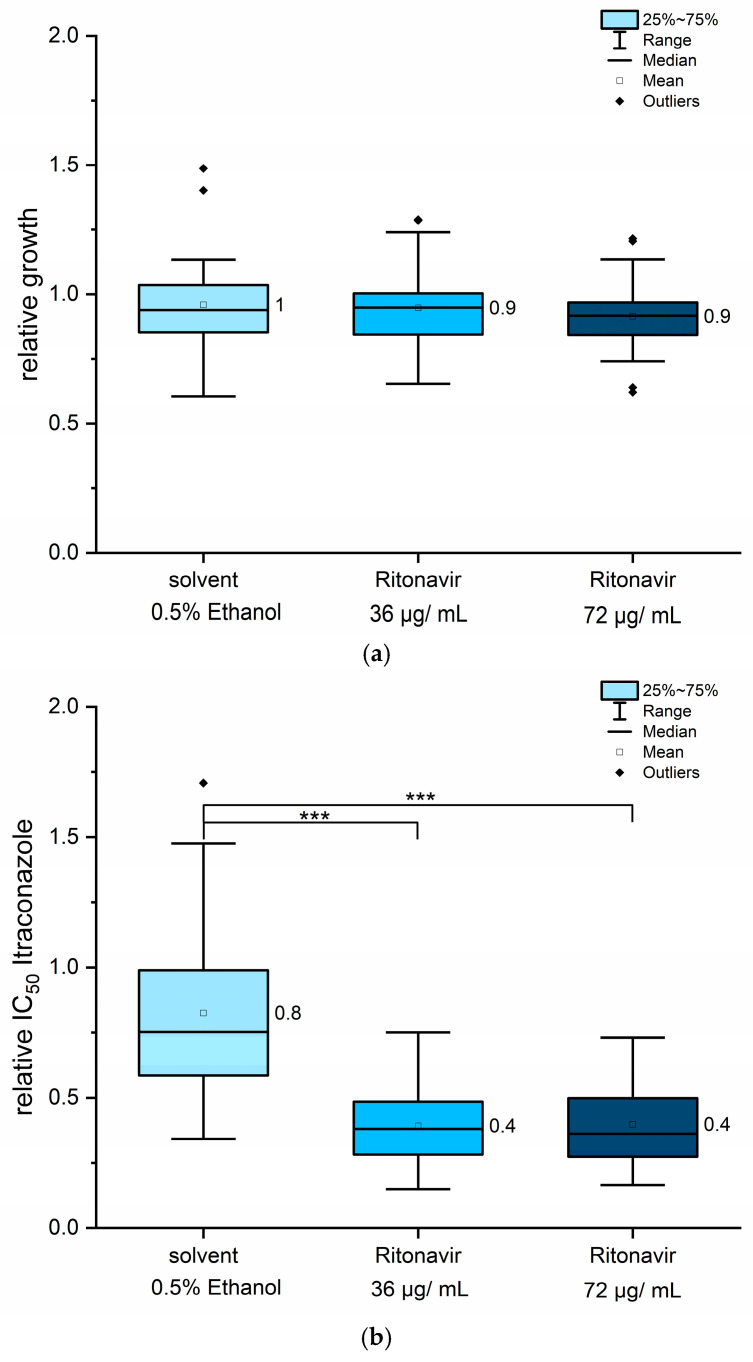
Ritonavir, when used in combination with itraconazole, reduced IC_50_ values by up to 50%. The effects of ritonavir on fungal growth are shown in panel (**a**), while relative IC_50_ values for itraconazole under co-treatment are presented in panel (**b**). All 12 strains were included in the analysis. Mean values are displayed next to each column. Statistical analysis was performed using pairwise Mann–Whitney U tests. Asymptotic significance levels (*p*) are indicated (*** *p* < 0.001).

In contrast to quinine hydrochloride, ritonavir is a hydrophobic compound and was therefore dissolved in ethanol. As ethanol can exert toxic effects on fungal cells, its potential influence on fungal growth was assessed as an additional control in all experiments. The final ethanol concentration was consistently maintained at 0.5% (*v*/*v*). The effects of ethanol alone or ritonavir alone (at 36 µg/mL and 72 µg/mL) on fungal growth were evaluated in the absence of itraconazole in each MLN assay for all strains. Neither 0.5% ethanol nor ritonavir in either concentration significantly affected fungal growth (Figure 4a), indicating that ritonavir alone has no intrinsic antifungal properties at the concentrations used. However, in combination with itraconazole, ritonavir led to an approximately 60% reduction in IC_50_ values of itraconazole (Figure 4b). The effect was observed at both 36 µg/mL and 72 µg/mL ritonavir, with no additional benefit observed at the higher concentration (Figure 4b). Interestingly, the solvent ethanol causes a modest reduction of about 20% in itraconazole IC_50_ values (Figure 4b), despite having no effect on growth kinetics itself (Figure 4a). This suggests that ethanol may induce physiological changes in fungal cells or influence physicochemical properties of itraconazole, for example, solubility, which influence drug sensitivity. After accounting for the ethanol effect, ritonavir itself appears responsible for approximately 50% of the observed IC_50_ reduction.

The strain-dependent response to ritonavir, as presented in Table 2 and in Table S3, did not correlate with any specific resistance genotype. For several strains, no solvent-associated reduction in IC_50_ values was observed (Table S3). For example, strains UKJ392/18, UKJ334/19, UKJ262/21, UKJ476/21, and UKJ1067/21 were unaffected by ethanol exposure (Table S3). Doubling the ritonavir concentration produced itraconazole IC_50_ values similar to those obtained with 36 µg/mL of ritonavir co-treatment, suggesting a saturation effect and indicating that the potentiating activity of ritonavir has an upper limit. IC_50_ values for itraconazole were derived from the inflection point of the respective logistic dose–response curves and were therefore less influenced by the slope of the curves. Since MIC_90_ values are more dependent on curve steepness, they are inherently more variable. To reduce the experimental variability, MIC_90_ values were obtained using identical pre-cultures and were averaged across two technical and two biological replicates. Notably, strain UKJ1708/17 exhibited MIC_90_ values approximately 28-fold higher than those of the susceptible strain UKJ1676/17 (Table 2). Strains UKJ1676/17 and UKJ1985/21 displayed the lowest MIC_90_ values and were considered susceptible isolates (Table 2). Interestingly, UKJ 1067/21 showed high MIC_90_ values, similar to strains with amplified *Erg11B* gene copies. Strain UKJ262/21 exhibited intermediate values, falling between the sensitive and resistant groups. Strains UKJ1067/21 and UKJ1985/21 were both isolated from the same patient, with the latter being recovered six months later after repeated terbinafine treatments [22]. Calculation of the fractional inhibitory concentration index (FICI) [46,47] was not feasible, because ritonavir alone does not exhibit sufficient antifungal activity. Instead, only the fractional inhibitory concentration (FIC) values for itraconazole were calculated (Table 2). FIC values for itraconazole ranged from 0.21 to 0.83, with most values clustering near 0.5 (Table 2), in agreement with the relative decrease in IC_50_ values observed (Figure 4b). Growth inhibition by ritonavir alone ranged from negligible to a maximum of 16% (Table 2). In contrast, reductions in MIC values for the combined treatments were consistently greater than the effects of ritonavir alone (Table 2). These findings indicate that co-administration of ritonavir and itraconazole resulted in synergistic interactions.

### 3.4. Sertraline Showed Antifungal Properties and Synergistic Effects in Combination with Itraconazole

**Figure 5:**
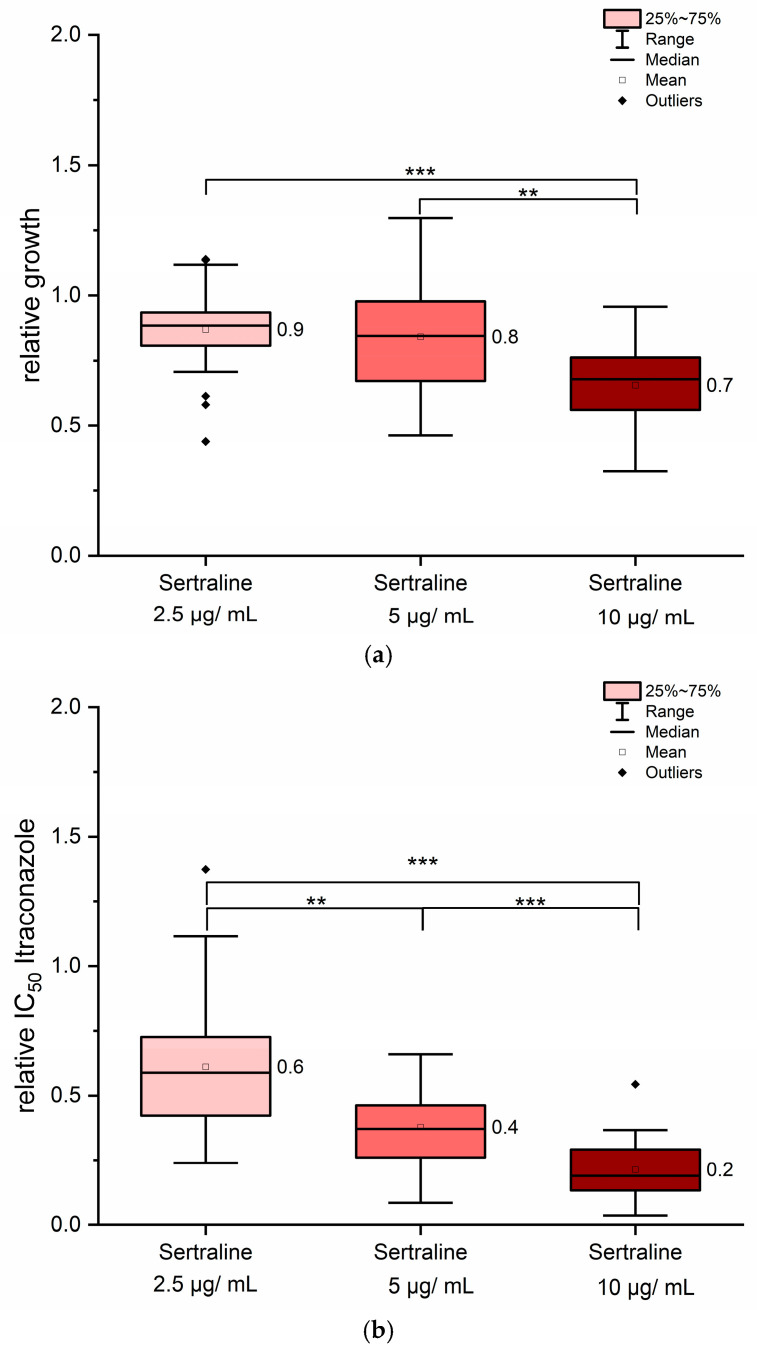
Sertraline exerted synergistic effects when combined with itraconazole. As shown in panel (**a**), sertraline alone inhibited fungal growth in a concentration-dependent manner. The ratio for itraconazole IC_50_ values in combination with sertraline is presented in panel (**b**). All 12 strains were included in the analysis. Mean values are indicated next to each column. Statistical significance was assessed using pairwise Mann–Whitney U tests. Asymptotic significance levels (*p*) are indicated (** *p* < 0.01; *** *p* < 0.001).

The most promising effects were observed with the combination of sertraline and itraconazole. In contrast to quinine hydrochloride and ritonavir, sertraline exhibited intrinsic antifungal activity, even in the absence of co-administration of itraconazole. To determine an appropriate concentration range, sertraline was evaluated for its standalone antifungal activity. A concentration of 20 µg/mL of sertraline resulted in complete growth inhibition in some strains. The solvent DMSO, used in a final concentration of 0.25% *v*/*v*, showed no significant influence on fungal growth. For combination treatments, sertraline concentrations between 2.5 µg/mL and 10 µg/mL were tested. At 10 µg/mL, sertraline alone caused significant growth reduction across nearly all tested strains (Figure 5a) but not below 75% of growth inhibition (Figure 5a). Therefore, MIC_90_ values of sertraline could be estimated above to be 10 µg/mL. In contrast, 2.5 µg/mL had no significant impact on fungal growth, while 5 µg/mL produced strain-dependent responses, suggesting intermediate sensitivity levels among isolates (Figure 5a). Although the lowest sertraline concentration (2.5 µg/mL) had no measurable antifungal effect on its own, its combination with itraconazole led to about 40% reduction in itraconazole IC_50_ values (Figure 5b). Doubling the sertraline concentration further reduced itraconazole IC_50_ values by approximately 60% and the highest sertraline concentration tested (10 µg/mL) yielded reductions of up to 80% (Figure 5b). These findings indicate a synergistic interaction between itraconazole and sertraline, enhancing antifungal efficacy beyond the additive effects of either compound alone.

The strains differed in their response to sertraline when co-administered with itraconazole, as summarized in Table S4. Nevertheless, no correlation was observed between specific genotypes and the sertraline-dependent response. Weaker effects of sertraline were noted for strains UKJ1676/17, UKJ1708/17, and UKJ334/17. In contrast, a pronounced sensitivity to sertraline was observed for UKJ392/18, UKJ421/18, UKJ893/19, and UKJ476/21 (Table S4). Interestingly, the greatest variability in strain-specific responses was observed at the highest sertraline concentration (10 µg/mL), with up to a four-fold difference in IC_50_ values for itraconazole. At the lowest sertraline concentration (2.5 µg/mL), variation between strains was limited to approximately two-fold. In contrast to ritonavir, no saturation effect was detected in the sertraline concentration range tested here; higher sertraline amounts consistently produced greater reductions in itraconazole IC_50_ values (Figure 5, Table S4). The highest MIC_90_ values were observed for strain UKJ1708/17, whereas the lowest values were found for UKJ1985/21 and UKJ 1676/17 (Table 2 and Table 3). Therefore, strain UKJ1708/17 exhibited approximately 22-fold higher MIC_90_ values compared to UKJ1676/17. Growth reduction by sertraline alone (10 µg/mL) ranged from 11% up to 53% across the tested isolates (Table 3). In contrast, reductions in MIC_90_ values observed for the combination of sertraline with antifungals were consistently much greater than those achieved with sertraline alone. An exception was strain UKJ334/19, for which the percentage reduction was comparable for both conditions (Table 3). Notably, combined antifungal treatment reduced MIC_90_ values by up to 90%, effectively bridging the gap between the most resistant isolates and the susceptible strains UKJ1676/17 and UKJ1985/21 (Table 3).

## 4. Discussion

The MLN-based method, adapted for antifungal susceptibility testing, proved suitable for detecting even subtle effects in fungal responses to the various drugs tested. The inclusion of single treatment controls for each substance in every assay eliminated influences related to pre-cultivation conditions and the physiological state of the isolates, an important requirement for reliably analyzing the impact of co-medicated compounds. While quinine hydrochloride showed only minor effects, both ritonavir and sertraline demonstrated clear synergistic interaction in combination with itraconazole. The decrease in relative IC_50_ values enabled the direct comparison between strains, regardless of their baseline susceptibility to different antifungal concentrations. Notably, the effects of ritonavir and sertraline were observed across both resistant and sensitive strains. Checkerboard dilution panels, commonly used to determine FICI values, often lack technical replicates and are therefore prone to variability and lower reproducibility [48]. Therefore, the inclusion of both technical and biological replicates in this study was essential, even though the dilution range was limited.

Multiple drug resistance (MDR) is frequently associated with the overexpression of ABC efflux transporters, which significantly impairs the efficacy of pharmaceutical interventions in humans. Quinine is considered a first-generation inhibitor of MDR transporters in humans [30]. Such first-generation inhibitors are often themselves substrates of the respective transporter, and their inhibitory properties were initially observed as off-target side effects [36]. Ritonavir functions as a substrate for the human ABC efflux transporter P-gp (MDR1) and the multidrug resistance-associated protein MRP1 [30]. Its role as a transport inhibitor has been demonstrated in studies investigating the uptake of technetium-labeled mebrofenin in humans [49]. Ritonavir also exhibits intrinsic antifungal activity, with a reported MIC_80_ of approximately 1 µg/mL (~1.4 mM) against the mycelial form of *Histoplasma capsulatum* [32]. Furthermore, the combination of ritonavir or saquinavir with itraconazol demonstrated synergistic antifungal effects against *H. capsulatum* [32]. In contrast, no antifungal activity of ritonavir alone was observed against *T. indotineae* strains in the present study, even at concentrations as high as 72 µg/mL. Nevertheless, ritonavir did exhibit a saturation effect when combined with itraconazole, with its potentiating effect reaching a plateau at about 50% IC_50_ reduction. In a recent study, lopinavir and ritonavir, when used alone, did not show antifungal activity against *Candida auris* strains [33]. However, in combination with various azoles, both demonstrated synergistic effects on the growth inhibition of *Candida auris* strains [33]. In this study, synergy was observed in nearly all *Candida auris* strains tested, 100% for lopinavir with itraconazole and 91% for ritonavir with itraconazole [39]. While synergistic interactions between ritonavir and itraconazole were also observed for *Trichophyton indotineae* strains in the present study, the overall effect was weaker.

In contrast, the combination of itraconazole with sertraline consistently demonstrated the most pronounced reductions in MIC_90_ values among all tested antifungal combinations in *T. indotineae* (Table 3). Synergistic interactions were reported when sertraline was combined with azoles against *Cryptococcus neoformans* [37], *Trichosporon asahii* [40], and *Coccidioides. immitis* [41]. Sertraline has also been shown to exhibit antifungal activity against *Trichophyton rubrum,* with synergistic effects noted in combination with caspofungin [42]. Antifungal susceptibility testing in that study followed the CLSI protocol [26], yielding MICs of 100 µg/mL with SG medium and 25 µg/mL with MOPS-buffered RPMI1640 medium for sertraline alone [42]. Caspofungin is not considered a first-line treatment for dermatomycosis, and MICs of 31–63 µg/mL for *T. rubrum,* depending on the growth medium, indicate relative low sensitivity [42].

Sertraline has been shown to influence the transcriptional activity of genes associated with the major facilitator superfamily (MFS) transporters and the C8-sterol isomerase involved in the ergosterol biosynthesis pathway [42]. Interestingly, synthetic sertraline derivatives also reduced ergosterol biosynthesis through the inhibition of the Δ5,6 desaturase [39]. The role of transporter expression on antifungal efflux in azole resistance was prominently shown for MDR3 in *T. rubrum* [20] and *T. indotineae* [18]. *Trichophyton mentagrophytes* isolates exhibiting terbinafine resistance in the absence of *Erg1* mutations were found to have increased transporter expression [21]. Transcriptome analyses of resistant *T. tonsurans* isolates further revealed upregulation of several transporter genes in response to antifungal exposure in resistant strains, indicating that efflux regulation is multifactorial and complex [50]. RNA-seq analysis of *T. rubrum* treated with sertraline elucidated altered expression patterns for several ergosterol biosynthesis genes and ABC transporter genes [51]. Additionally, the ergosterol content of *T. rubrum* was significantly reduced when treated with sub-inhibitory concentrations of sertraline [51]. These findings and the results of the present study support the hypothesis that sertraline enhances azole efficacy by targeting components of the ergosterol biosynthesis pathway, thereby explaining the consistent synergistic effects observed in combination therapies. Expression analyses demonstrated that *T. indotineae* upregulated the efflux transporter MDR3 in response to fluconazole or voriconazole, but not to itraconazole, suggesting an impaired sensing mechanism for the latter [18]. A novel itraconazole formulation with improved bioavailability (SUBA-itraconazole) enables dose reduction while enhancing clinical antifungal efficacy [52].

A key limitation of this study is that the observed effects of sertraline were restricted to in vitro conditions, and in vivo studies for validation are required before clinical applications can be considered. Reported serum concentrations of sertraline [53] may be too low to achieve systemic antifungal efficacy, and its suitability for topical administration remains uncertain. Future studies are needed to determine whether sertraline can penetrate the skin sufficiently to support its use in the treatment of dermatomycoses.

## 5. Conclusions

This study demonstrates that co-treatment with ritonavir or sertraline significantly enhances the antifungal efficacy of itraconazole against *Trichophyton indotineae*, including azole-resistant strains. The observed reduction in IC_50_ and MIC_90_ values supports the interpretation of pharmacological synergy. Together, these findings suggest that the adjunctive use of repurposed drugs such as ritonavir and sertraline may offer a promising strategy to overcome azole resistance in dermatophytes. Further in vivo studies and mechanistic analyses are warranted to validate these results and support clinical translation.

## Figures and Tables

**Table 1:** Summary of the *Trichophyton indotineae* strains included in this study and their characteristic features.

UKJ Number	Collection Synonyms	*Erg1* Amino Acid Exchanges	*Erg11B* Amino Acid Exchanges	*Erg11B* Amplification Type	Source, Cited
_UKJ_1676/17	_TIMM_20114	Ala448Thr	Wild type	No amplificates	[9,15,17]
_UKJ_1687/17	_TIMM_20118	Phe397Leu	Gly443Glu	Type I	[9,15,17]
_UKJ_1708/17		Ala448Thr	Wild type	Type II	[9,15,17]
_UKJ_392/18	_TIMM_20117, 200087/18	Ala448Thr	Wild type	Type II	[9,15,17]
_UKJ_421/18	_TIMM_20119, 200123/18	Phe397Leu	Gly443Glu	Type I	[9,15,17]
_UKJ_334/19	_TIMM_201120, 250082/18	Ala448Thr	Wild type	Type II	[9,17]
_UKJ_336/19	_TIMM_201121, 250084/18	Phe397Leu, Ala448Thr	Wild type	Type II	[9,17]
_UKJ_893/19	_TIMM_201123, 600097/19	Ala448Thr	Wild type	Type II	[9,17]
_UKJ_262/21		Ala448Thr	Tyr444His	No amplificates	[14,18]
_UKJ_476/21		Ala448Thr	Wild type	Type II	[14,18]
_UKJ_1067/21		Wild type	Ala230Thr, Tyr444His	No amplificates	[14,18,22]
_UKJ_1985/21		Wild type	Ala230Thr, Tyr444His	No amplificates	[18,22]

**Table 2:** Synergistic effects of ritonavir. MIC_90_ values for itraconazole (ITZ) alone were compared to those obtained in combination with 36 µg/mL of ritonavir to form the fractional inhibitory concentration for itraconazole (FIC ITZ). Mean values derived from two technical replicates and two biological replicates are shown, ordered according to the MIC_90_ values for itraconazole alone.

Strain	MIC_90_ ITZ Alone µg/mL	MIC_90_ ITZ with Ritonavir µg/mL	FIC ITZ	% Reduction MIC_combined_	% Growth Reduction of Ritonavir Alone
_UKJ_1708/17	0.069	0.057	0.83	17	3
_UKJ_336/19	0.028	0.0058	0.21	79	14
_UKJ_421/18	0.024	0.0091	0.38	62	4
_UKJ_893/19	0.024	0.0072	0.30	70	6
_UKJ_476/21	0.023	0.015	0.67	33	12
_UKJ_392/18	0.022	0.0087	0.40	60	0
_UKJ_1067/21	0.017	0.0090	0.53	47	3
_UKJ_1687/18	0.013	0.0072	0.53	47	3
_UKJ_334/19	0.012	0.0053	0.43	57	1
_UKJ_262/21	0.0095	0.0040	0.43	57	14
_UKJ_1985/21	0.0032	0.0012	0.36	64	14
_UKJ_1676/17	0.0025	0.0013	0.51	49	16

**Table 3:** Synergistic effect of sertraline. MIC_90_ values for itraconazole alone were compared with MIC_90_ values combined with 10 µg/mL of sertraline. Mean values derived from two technical replicates and two biological replicates are shown, ordered according to the MIC_90_ values for itraconazole alone.

Strain	MIC_90_ ITZ Alone µg/mL	MIC_90_ ITZ with Sertraline µg/mL	FIC ITZ	% Reduction MIC_combined_	% Growth Reduction of Sertraline Alone
_UKJ_1708/17	0.061	0.026	0.42	58	11
_UKJ_421/18	0.047	0.0056	0.12	88	33
_UKJ_893/19	0.033	0.0039	0.12	88	25
_UKJ_476/21	0.028	0.011	0.40	60	28
_UKJ_1687/18	0.024	0.0025	0.11	90	30
_UKJ_334/19	0.018	0.0076	0.42	58	53
_UKJ_336/19	0.017	0.0053	0.32	68	40
_UKJ_1067/21	0.015	0.0025	0.17	83	49
_UKJ_392/18	0.012	0.0023	0.19	81	26
_UKJ_262/21	0.0105	0.0028	0.26	74	24
_UKJ_1676/17	0.0032	0.0011	0.36	64	33
_UKJ_1985/21	0.0028	0.00039	0.14	86	42

## Data Availability

The original contributions presented in this study are included in the article/Supplementary Material. Further inquiries can be directed to the corresponding author.

## Supplementary Materials

The following supporting information can be downloaded at https://www.mdpi.com/article/10.3390/jof11100698/s1; Table S1: *Trichophyton indotineae* strains and GenBank Accession Number (Acc. No.) entries; Table S2: Strain specific relative IC_50_ values for itraconazole after co-treatment with quinine hydrochloride; Table S3: Strain dependent reduction of IC_50_ values for itraconazole effect of upon co-treatment with ritonavir and ethanol alone as solvent control; Table S4: Strain dependent reduction of IC_50_ values for itraconazole effect upon co-treatment with sertraline.

## Abbreviations

The following abbreviations are used in this manuscript:

CFU	Colony-forming units
DMSO	Dimethyl sulfoxide
FICI	Fractional inhibitory concentration index
IC	Inhibitory concentration
ITZ	Itraconazole
MDR	Multiple drug resistance
MFS	Major facilitator superfamily
MIC	Minimal inhibitory concentration
MLN	Microplate laser nephelometry
MOPS	3-(N-morpholino)propane sulfonic acid
RPMI	Roswell Park Memorial Institute
SG	Sabouraud-glucose medium
