# Supplementary material for: Co-Treatment with Ritonavir or Sertraline Enhances Itraconazole Efficacy Against Azole-Resistant Trichophyton indotineae Isolates
Source: J Fungi (Basel). 2025 Sep 25;11(10):698. doi: 10.3390/jof11100698 (PMC12564935; doi:10.3390/jof11100698)
Supplement: Supplementary file 1 [file jof-11-00698-s001.zip › jof-3848830-supplementary.pdf]

Table S1. *Trichophyton indotineae* strains and GenBank Accession Number (Acc. No.) entries. *T. indotineae* strains from 2017-2019 were collected and analyzed for ITS and *Erg1* in a previous study [9]. Part of these strains were shared with other research groups, who used these isolates for genome analyses [15, 17]. For strains UKJ1708/17 and UKJ334/19 DNA sequences of *Erg1* and *Erg11B* showed in BLAST hits 100% of sequence identity (id.) to GenBank Acc. No. of *T. indotineae* strain UKJ476/21 [14] used as reference.

UKJ number	Collection synonyms	*Erg1* GenBank Acc. No.	*Erg11B* GenBank Acc. No.	Genome assembly	Source, cited
UKJ1676/17	TIMM20114			GCA_023065905	[9, 15, 17]
UKJ1687/17	TIMM20118			GCA_023065865	[9, 15, 17]
UKJ1708/17		Id. MZ636379	Id. MZ636375		[9, 15, 17]
UKJ392/18	TIMM20117, 200087/18			GCA_023065795	[9, 15, 17]
UKJ421/18	TIMM20119, 200123/18			GCA_023065815	[9, 15, 17]
UKJ334/19	TIMM201120, 250082/18	Id. MZ636379	Id. MZ636375		[9, 17]
UKJ336/19	TIMM201121, 250084/18			GCA_032157385	[9, 17]
UKJ893/19	TIMM201123, 600097/19			GCA_032157405	[9, 17]
UKJ262/21		MZ636378	MZ636376		[14, 18]
UKJ476/21		MZ636379	MZ636375		[14, 18]
UKJ1067/21		OK572480	OK572479		[14, 18, 22]
UKJ1985/21		PP549428	PP549426		[18, 22]

Table S2. Strain specific relative IC_{50} values for itraconazole after co-treatment with quinine hydrochloride.

Strain	Quinine HCl 2.5 µg/ mL	Quinine HCl 5 µg/ mL	Quinine HCl 10 µg/ mL
UKJ1676/17	0.91	0.82	1.04
UKJ1687/17	0.62	0.61	0.70
UKJ1708/17	0.87	0.89	0.84
UKJ392/18	0.93	0.94	0.76
UKJ262/21	0.56	0.68	0.80
UKJ476/21	1.01	0.89	0.97
UKJ1067/21	0.79	0.56	0.52
UKJ1985/21	0.93	0.77	0.71

Table S3. Strain dependent reduction of IC_{50} values for itraconazole effect of upon co-treatment with ritonavir and ethanol alone as solvent control. Relative reduction of IC_{50} values for itraconazole were shown as mean values with standard deviation.

Strain	Ethanol (solvent) 0.5 % w/v	Ritonavir 50 mM	Ritonavir 100 mM
UKJ1676/17	0.49 ± 0.05	0.34 ± 0.12	0.37 ± 0.11
UKJ1687/17	0.76 ± 0.05	0.63 ± 0.12	0.69 ± 0.02
UKJ1708/17	0.59 ± 0.003	0.43 ± 0.04	0.26 ± 0.09

UKJ392/18	1.04 ± 0.07	0.62 ± 0.05	0.47 ± 0.19
UKJ421/18	0.70 ± 0.04	0.26 ± 0.10	0.38 ± 0.09
UKJ334/19	0.96 ± 0.11	0.29 ± 0.03	0.34 ± 0.09
UKJ336/19	0.52 ± 0.18	0.29 ± 0.14	0.37 ± 0.18
UKJ893/19	0.71 ± 0.19	0.38 ± 0.10	0.45 ± 0.08
UKJ262/21	0.88 ± 0.30	0.41 ± 0.12	0.38 ± 0.09
UKJ476/21	1.05 ± 0.04	0.47 ± 0.02	0.50 ± 0.23
UKJ1067/21	0.93 ± 0.40	0.35 ± 0.05	0.33 ± 0.04
UKJ1985/21	0.79 ± 0.13	0.31 ± 0.01	0.31 ± 0.07

Table S4. Strain dependent reduction of IC_{50} values for itraconazole effect upon co-treatment with sertraline. Relative reduction of IC_{50} values for itraconazole were shown as mean values with standard deviation.

Strain	Sertraline 2.5 µg/ mL	Sertraline 5 µg/ mL	Sertraline 10 µg/ mL
UKJ1676/17	0.64 ± 0.13	0.46 ± 0.21	0.19 ± 0.04
UKJ1687/17	0.47 ± 0.20	0.22 ± 0.01	0.15 ± 0.01
UKJ1708/17	0.69 ± 0.02	0.51 ± 0.08	0.21 ± 0.06
UKJ392/18	0.34 ± 0.01	0.16 ± 0.04	0.09 ± 0.03
UKJ421/18	0.37 ± 0.03	0.22 ± 0.01	0.13 ± 0.02
UKJ334/19	0.75 ± 0.09	0.51 ± 0.02	0.16 ± 0.03
UKJ336/19	0.54 ± 0.24	0.39 ± 0.08	0.17 ± 0.04
UKJ893/19	0.38 ± 0.01	0.24 ± 0.03	0.12 ± 0.02
UKJ262/21	0.62 ± 0.08	0.40 ± 0.02	0.11 ± 0.02
UKJ476/21	0.40 ± 0.14	0.23 ± 0.17	0.08 ± 0.05
UKJ1067/21	0.57 ± 0.04	0.29 ± 0.01	0.05 ± 0.02
UKJ1985/21	0.41 ± 0.05	0.23 ± 0.04	0.13 ± 0.06
